# Effect of *Rhizoma coptidis* (Huang Lian) on Treating Diabetes Mellitus

**DOI:** 10.1155/2015/921416

**Published:** 2015-10-05

**Authors:** Bing Pang, Xiao-Tong Yu, Qiang Zhou, Tian-Yu Zhao, Han Wang, Cheng-Juan Gu, Xiao-Lin Tong

**Affiliations:** ^1^Department of Endocrinology, Guang'anmen Hospital of China, Academy of Chinese Medical Sciences, Beijing 100054, China; ^2^College of Acupuncture, Beijing University of Chinese Medicine, Beijing 100029, China; ^3^Department of Digestion, Beijing Hospital of Traditional Chinese Medicine, Capital University of Medicine Sciences, Beijing 100010, China

## Abstract

The rapidly increasing diabetes mellitus (DM) is becoming a major public health issue globally; considerable progress has been made in the field of western hypoglycemic drug and insulin, but some shortages still exist. As one of the most important parts in complementary and alternative therapies, traditional Chinese medicine (TCM) performs a good clinical practice and is showing a bright future in the treatment of DM. TCM therapy has certain advantages of less toxicity and/or side effects, and Chinese herbal medicine which usually contains various active ingredients could provide multiple therapeutic effects. Huang Lian (*Rhizoma coptidis, RC*) is a herb frequently used in many traditional formulas for properties of “clearing damp-heat, quenching fire, and counteracting poison” in Asia for centuries. In this review, we summarize the application of *RC* in the treatment of DM from two aspects of contents. Firstly, theoretical principles are explained, including the properties and related records about *RC* in ancient references and modern pharmacological researches and pharmacokinetics on *RC* and its active components. Secondly, the clinical application of *RC* is mainly reviewed, such as applicable stage and syndrome, the reasonable dose range, the preparation formulations, and the toxicity and/or side effects and solutions to its adverse actions. This review provides scientific evidence about the effective components, pharmacological researches, and toxicity of *RC*, as well as introducing traditional Chinese medical theory and clinical experience, in order to guide clinician to use *RC* more suitably and reasonably in the clinical practice.

## 1. Introduction

Diabetes mellitus (DM) is a metabolic disorder in the endocrine system due to either an absolute deficiency in insulin secretion or reduction in the biological effectiveness of insulin. The global prevalence of DM among adults aged 20–79 years was 8.3% in 2013 [1]. As one of the largest developing countries, China has the biggest population of DM—92.4 million, which accounts for 9.7% of adult population [2]. In addition, 148.2 million adults (15.5%) have prediabetes [2]. DM is the 7th leading cause of mortality in the world, which can substantially result in a huge burden in terms of renal failure, nontraumatic lower-limb amputations, and newly diagnosed retinopathy and represent a major public health issue [3]. Type 2 diabetes mellitus (T2DM) is the predominant form of DM and accounts for 90–95% of diabetic people globally, due to an increased number of elderly people and a greater prevalence of obesity and sedentary lifestyle [4, 5]. Management of T2DM is still a challenge; currently, the standard therapy for T2DM includes diet, exercise, use of oral hypoglycemic drugs, and/or subcutaneous insulin injections [6]. These treatment methods frequently have side effects, such as weight gain, bone loss, and increased risk of cardiovascular events; treatment is costly as well, since T2DM is a chronic disease and long-term treatment is necessary [4–6]. In traditional Chinese medicine (TCM), DM may fall under the categories of “Xiaokezheng”. It is characterized by excessive fluid drinking, excessive food-consumption, excessive urination, and weight loss; all of these symptoms are commonly called “three excesses and one loss” [7]. The main pathogenesis of “Xiaokezheng” lies in yin deficiency leading to endogenous dryness-heat in the body, and blood stasis and phlegm retention often present. If prolonged yin deficiency impairs yang, this will result in dual deficiency of qi and yin as well as dual deficiency of yin and yang. Therefore, the main TCM treatment methods for Xiaokezheng are invigorating qi, nourishing yin, clearing away the heat, and promoting fluid production [3, 8, 9]. Famous formulas including Bai Hu Jia Renshen Tang, Jin Gui Shen Qi Wan, and Yuye Tang are widely used [3, 8–10]. TCM has a long history of more than 2,000 years to treat T2DM in China [11]. Treatment by TCM has certain advantages of less toxicity and/or side effects, and herbal medicine which usually contains various active components could provide multiple therapeutic effects on multiple targets, including enhancement of insulin sensitivity, stimulation of insulin secretion, or reduction of carbohydrate absorption [7, 12, 13]. There are 86 herbal medicines often used in the traditional Chinese formulas for T2DM and its complications; of these,* RC* is widely used for treatment of T2DM and its complications [12]. It is the rhizome of* Coptis chinensis* Franch that belongs to the Ranunculaceae family, which is recorded in the Chinese Pharmacopeia with the Chinese name of Huang Lian. It could clear the damp-heat, quench the fire, and counteract the poison, which was called as a holy herb to treat Xiaokezheng by Liu He-Jian in the Jin and Yuan Period [14]. In the Taiping Shenghui Formulary (*Tài Píng Shèng Huì Fāng*) of Song Dynasty,* RC *ranked in the first three among the frequently used ten medicines in the 177 prescriptions for Xiaokezheng [15]. Recent studies have indicated that* RC* possesses multispectrum therapeutic activities, including antihyperglycemia, antihyperlipidemia, antihypertension, anti-inflammatory, and antioxidant effects [12, 16–18]. In this review, we summarize the application of* RC *from two aspects of contents, including theoretical principles and the clinical practice of* RC* in both English and Chinese search engines, in order to guide clinician to use* RC* more suitably and reasonably in the clinical practice.

## 2. Theoretical Principles of Using* RC*

### 2.1. Properties of* RC *and Records in Ancient References

The use of* RC *in the treatment of DM is, first, to clear the damp-heat, quench the fire, and counteract the poison and, second, to lower the blood glucose. The combination of medicine properties and pharmacology ensures the good therapeutic effects [19, 20].* RC* is a herb of bitter flavor and cold property, entering channels of heart, spleen, stomach, gallbladder, and large intestine. It could clear away the excess heat, removing dampness and eliminating toxins according to traditional Chinese pharmacology [21]. The Supplementary Records of Famous Physicians (*Míng Yī Bié Lù*) in Wei-Jin Period firstly recorded the “treat Xiaokezheng” function of* RC*. The Newly Revised Herbal Foundation (*Xīn Xīu Bĕn Căo*) recorded that “*RC* in Sichuan had a large size and the most bitter flavor, which was the best for treatment of Xiaokezheng.” During Ming and Qing Dynasties, the Compendium of Materia Medica (*Bĕn Căo Gāng Mù*) written by Li Shi-Zhen, recorded that “*RC *was mainly used to deplete thirst and treat copious urine.” Main medicinal processing methods included (1) making honey pills; (2) steaming with wine, soaking with wine, or decocting with water; and (3) processing with wax gourd [15].

### 2.2. Modern Pharmacological Researches on Berberine and Other Alkaloids

*RC* was mainly composed of a diversity of alkaloids, including berberine (6.88% to 13.64%), palmatine (1.28% to 2.12%), jateorrhizine (0.77% to 1.32%), coptisine (0.42% to 0.85%), epiberberine (0.42% to 0.92%), worenine, and magnoflorine, all of which are considered to be its active components [22]. Berberine (BBR), an isoquinoline alkaloid, is the major active component of* RC*. Modern pharmacological researches have showed multiple mechanisms of BBR to lower blood glucose, such as improvement of insulin sensitivity, increase of insulin secretion [23], promotion of intestinal glucagon-like protein-1 (GLP-1) secretion [24], inhibition of hepatic gluconeogenesis [25], induction of glycolysis in peripheral tissues [26], promotion of antioxidant activities [16, 17], regulation of lipid disorders [27], and modulation of the gut microbiota [28, 29]. Lee et al. [30] showed that BBR improved insulin sensitivity and increased insulin secretion possibly through activating adenosine monophosphate-activated protein kinase pathway (AMPK) activity. Zhou et al. [31] showed that BBR modulated glycolipid metabolism possibly through increasing peroxisome proliferator-activated receptors (PPARs) PPAR*α*/*δ* expression and reducing PPAR*γ* expression in liver. Kong et al. [32, 33] found that BBR could increase the expression of the low-density lipoprotein receptor (LDLR) through an extracellular signal–regulated kinase (ERK)—dependent way. Moreover, BBR may exert antidiabetes effects via regulating gut microbiota [28]. Xie et al. [29] showed that BBR significantly reduced the number of harmful microbiota and increased the number of beneficial ones in the feces of high-fat diet-fed (HFD) mice, which may have a relationship with the glucose-lowering and lipid-lowering effects.

In addition to BBR, coptisine (COP), palmatine (PAL), and jatrorrhizine (JAT) were generally considered as the main bioactive components in* RC* [34]. Coptisine (COP), an isoquinoline alkaloid isolated from* RC*, possesses evident pharmacological activities against diabetic complications-related symptoms, including hypoglycemic [35], antiradical, antioxidant [36], and antibiotic effects [37]. Jiang et al. showed stronger hypoglycemic capability of COP in vitro compared with JAT and epiberberine but relatively weaker than BBR [38]. Jung et al. indicated that COP had inhibitory activities against aldose reductase, which may be important way to treat DM and diabetic complications [35]. Yokozawa et al. reported that COP was the most effective for protecting oxidative stress [17]. In another study, COP significantly decreased the levels of blood lipid in high-fat and high-cholesterol diet mice (HFHC). Results demonstrated that a high dosage of COP (70.05 mg/kg) could inhibit cholesterol synthesis via suppressing the 3-hydroxy-3-methyl-glutaryl-CoA reductase (HMGCR) expression, as well as promoting the use and excretion of cholesterol via upregulating LDLR and cholesterol 7*α*-hydroxylase (CYP7A1) expression [39]. Some researchers investigated that COP exerted the protective effects on myocardial ischemia and reperfusion (I/R) damage in rat, which may relate to its strong antioxidant activity. COP also decreased the proinflammatory cytokines, such as interleukin- (IL-)1*β*, IL-6, and tumor necrosis factor-*α* (TNF-*α*), by inhibiting the rhodopsin (Rho)/Rho-kinase (ROCK) pathway [40, 41]. PAL is also a main isoquinoline alkaloid isolated from* RC*, which has been used in the treatment of hypertension, inflammation, jaundice, dysentery, cardiovascular diseases, and liver-related diseases [42, 43]. PAL also had therapeutic effects on related symptoms of diabetic complications due to its hypoglycemic, hypolipidemic, and cardiovascular protective effects. Yuan et al. [18] reported that PAL possesses hypoglycemic and hypocholesterolemic activities. A PAL-derivative, namely, 11-hydroxypalmatine, was evaluated for the hypoglycemic activity. The PAL-derivative was administered to alloxan-induced diabetic mice at doses of 25, 50, and 100 mg/kg orally. The blood glucose significantly decreased by 52% after treatment with the PAL-derivative compared with the positive control glibenclamide (54%) and the diabetic control (27%) [44]. PAL was considered as a potential agent for lowering the blood lipids. The mechanisms may relate to upregulation of LDLR and CYP7A1 mRNA and protein expression [45]. JAT is one of the major bioactive components isolated from* RC*. Some studies have demonstrated the hypoglycemic activity of JAT in alloxan-induced mice [46]. Wang et al. showed the effect of JAT on glucose uptake. JAT at different concentrations (265.65, 53.75, 10.75, 2.15, and 0.45 *μ*mol/L) was administered to 3T3-L1 adipocytes for different periods (12, 24, 48, and 72 h), indicating that the optimal active concentration of JAT was 0.45 *μ*mol/L and the preferable reaction time was 48 hours. Moreover, JAT can promote the fatty acid oxidation in 3T3-L1 adipocyte, which may be attributable to the upregulation of PPAR*α* and PPAR *β* levels [47]. Wu et al. showed that JAT had a strong hypolipidemic effect in a dose-dependent way mainly through upregulating the mRNA and protein expression of LDLR and CYP7A1 [48]. Epiberberine is also a natural bioactive protoberberine alkaloid, which showed the mild hypoglycemic effects and low cytotoxicity of* RC* extract in HepG2 cells [34]. Yuan et al. [18] reported that BBR, PAL, and JAT were identified as active components in* RC* extract to lower the blood glucose and lipids, which were marked by a dose-dependent manner; when rats were given* RC* extract orally at a dose of 0.5 g/kg. day for 3 weeks, the blood glucose reduced by 58%. Yokozawa et al. [17] reported that COP, PAL, magnoflorine, and epiberberine might contribute to the protective effects of* RC* on oxidative stress through inhibition of cellular peroxynitrite generation.

The alkaloids isolated from* RC *may have beneficial effects on diabetic complications and DM due to the inhibitory activities against *α*-glucosidase or aldose reductase [49].* RC* extract had the *α*-glucosidase inhibitory activity with the half maximal inhibitory (IC50) value at 3.528 mg mL(-1), which could be effective for treating DM, and the alkaloids were main components that inhibited *α*-glucosidase activity in* RC* extract [50]. Zhou et al. screened for *α*-glucosidase inhibitors, five components in the* RC* extract were found, and their structures were identified by electrospray ionization tandem mass spectrometry (ESI-MS) to be COP, epiberberine, JAT, BBR, and PAL [51]. Aldose reductase (AR) is the enzyme that leads to conversion of glucose to sorbitol, and its increased activity signifies in the development of long-term complications of DM [52]. Jung et al. [35] evaluated the inhibitory activities of the alkaloids from* RC *AR for the treatment of diabetic complications. Results showed that epiberberine, COP, and groenlandicine exhibited moderate inhibitory effects with IC(50) values of 100.1, 118.4, and 140.1 microM for rat lens aldose reductase (RLAR) and 168.1, 187.3, and 154.2 microM for human recombinant aldose reductase (HRAR), but BBR and PAL did not exhibit AR inhibitory effects at a higher concentration of 50 microg/ml, indicating that the presence of the dioxymethylene group in the D ring and the oxidized form of the dioxymethylene group in the A ring played important roles in inhibiting AR.* RC* and contained alkaloids therein had therapeutic effects on DM and its complications. Kwon et al. [53] showed the protective effect of* RC* on the cytotoxicity of pancreatic beta-cells; the action mechanism may relate to protecting apoptosis and necrosis through the inhibition of Deltapsim disruption, indicating that* RC *may be effective for preventing type 1 diabetes mellitus (T1DM).

*RC* extracts may be more effective than its single alkaloid; the mechanisms are related to the fact that different components may not only regulate targets in multiple pathways, and therefore enhancing pharmacological potency in a synergistic way, but also regulate the enzymes and transporters that are involved in hepatic and intestinal metabolism to improve oral drug bioavailability [54]. Fu et al. [46] compared the hypoglycemic activities of JAT, BBR,* RC* decoction, and compounds-mimic prescription (BBR-JAT) on blood glucose level in mice. Data suggested that* RC* decoction showed the most significant hypoglycemic activity. JAT also possessed the effect of decreasing blood glucose, which was less than that of BBR at the same dose. There was no significant difference between BBR-JAT and BBR (*P* > 0.05). The results showed that* RC *decoctionwas more effective than its single components BBR and JAT, indicating that other hypoglycemic components existed. Liu et al. [55] suggested that area under curve (AUC) and peak concentration (*C*_max⁡_) of BBR significantly increased in rats receiving* RC* extract compared with those receiving the pure BBR, indicating that* RC* extract showed better hypoglycemic activity than pure BBR. Liu et al. demonstrated the different metabolic interaction between the active components (BBR, COP, PAL, and JAT) of* RC* in human liver microsomes by HPLC. COP showed inhibition against the formation of the two metabolites of BBR with IC50 values of 6.5 and 8.3 *μ*M, respectively, which indicated the strongest inhibition toward BBR metabolism. BBR suggested a weak inhibition against the production of COP metabolite with an IC50 value of 115 *μ*M. PAL and JAT showed the weaker inhibitions against the formation of the metabolites of BBR and had little inhibitory effect on the formation of COP metabolite. BBR, COP, and JAT showed no inhibitory effect on the generation of PAL metabolite with an IC50 of more than 200 *μ*M [55]. Zhu et al. [56] compared the hypoglycemic activity of* RC* alkaloids (BBR, JAP, PAL, and COP) in HepG2 cell through measuring the glucose consumption and effect of cell vitality. Results indicated the alkaloids which ranged from 0.2 to 5 mg/L had the hypoglycemic activity, and BBR showed then cytotoxicity at the doge of 5 mg/L. Besides, composite alkaloids showed no cytotoxicity, but higher hypoglycemic potency compared with pure alkaloid, which may be achieved by synergistic interaction between alkaloids. Similar results were also demonstrated by another study [57]. Whether the hypoglycemic activity is best exerted synergically in a formula or multicomponent or independently as an active component remains to be investigated. The possible hypoglycemic effects and mechanisms of the other components of* RC* and the interactions among its various components are still needed to be demonstrated in future researches.

### 2.3. Pharmacokinetics of* RC*

The pharmacokinetic properties and potential herb-drug interactions found with* RC *alkaloids have been demonstrated by several studies. Generally, the systemic exposures of the alkaloids are extremely low after oral administration. The alkaloids may present their systemic effects through generated metabolites and/or the tissue distributed alkaloids themselves or through modulating effectors in the gut [58]. Yu et al. [59] investigated the pharmacokinetics of BBR, PAL, COP, epiberberine, and jatrorrhizine from* RC* in diabetic rats.* RC* extract (1.3 g/kg) was administered by oral gavage to the control rats and the diabetic rats induced by 6-week injection of streptozotocin. Blood samples (300 *μ*L) were obtained from the ocular fundus vein before dosing and subsequently at selected intervals of 0.25, 0.5, 1.0, 1.5, 2, 2.5, 3, 4, 6, 8, 12, and 24 h following administration. The concentrations of five types of alkaloids were determined. Compared to those in the control, there existed 170% to 330% increases in *C*_max⁡_ and 150% to 350% increases in AUC_0–24_ of the five types of alkaloids, the bioavailability of the five protoberberine type alkaloids of* RC* extract enhanced in diabetic rats, which may potentially reach therapeutic concentrations and exert pharmacological effects in the body. Therefore, DM is responsible for the enhancements in the bioavailability of the five protoberberine type alkaloids of* RC *extract in rats, perhaps due to the pathological changes in absorption, distribution, metabolism, and excretion. In the previous study, Yu et al. showed that, after oral administration of* RC* extract, BBR, PAL, COP, epiberberine, and jatrorrhizine in 6-week streptozotocin- (STZ-) induced diabetic rats acquired a higher exposure. Subsequently, they demonstrated that the function and expression of intestinal P-glycoprotein (P-GP) were downregulated in STZ-induced diabetic rats, which contributed to the increased exposure of the five protoberberine alkaloids [60]. BBR, PAL, and jateorrhizine exhibited similar plasma concentration-time profiles under the same administration methods, which probably resulted from the similarity of their molecular structures, which contributed to their similar disposition process in vivo. A pharmacokinetic study suggested that *C*_max⁡_ of BBR, PAL, and jatrorrhizine was 78.42 ± 12.19, 82.09  ± 17.44, and 55.35 ± 8.90 *μ*g/L, respectively. All *T*_max⁡_ of BBR, PAL, and jatrorrhizine was 0.75 ± 0.11 [61]. The quantification of three alkaloids (BBR, PAL, and COP) was investigated in the pharmacokinetics of the alkaloids from Xiexin Decoction in rats. A high-performance liquid chromatographic method was used. Results indicated that the linear ranges of the calibration curves were 1.6–160 ng/ml for all three alkaloids. The within-batch accuracy was 88.6–107.8% for BBR, 88.4–110.1% for PAL, and 90.4–108.3% for COP; the between-batch accuracy was 94.3–100.6% for BBR, 99.3–100.3% for COP, and 93.7–100.0% for PAL; the within-batch and between-batch precisions were <or = 10.1% and <or = 11.1%, respectively [62]. In a pharmacokinetic study, Xue et al. investigated the interaction of magnoflorine with the rest of compounds in* RC*. The rats were not only administered with magnoflorine orally (15, 30, and 60 mg/kg) and intravenously (10 mg/kg), but also administered with* RC *decoction (equivalent to 30 mg/kg of magnoflorine) intragastrically. Data suggested that magnoflorine possessed lower bioavailability and faster absorption and elimination. However, when magnoflorine was administered in* RC* decoction, pharmacokinetic parameters significantly altered (*P* < 0.05). Oral gavage of* RC* decoction decreased the absorption and elimination rates of magnoflorine, indicating that the pharmacokinetic interactions existed between magnoflorine and other components in* RC* [63]. Guo and Zhao [64] used different dosages of BBR group (40, 80, and 120 mg/kg, resp.) to treat diabetic mice, compared with gliclazide group (5 mg/kg). All the indicators showed no significant difference between groups (*P* > 0.05). When mice of each group were administrated drugs for 60 days, although hypoglycemic activity of BBR appeared slower than those of gliclazide, BBR improved the glucose tolerance and increased the level of insulin, which showed obvious dose-effect relationship.

## 3. The Clinical Practice of Using* RC*

### 3.1. Applicable Stage and Syndrome

Wu and Wei [65] observed efficacy of BBR in treating T2DM. Seventy-two cases were assigned to obese group and nonobese group; BBR (0.02 mg/kg) was administrated orally for 8 to 10 weeks. Results showed that insulin resistance and body mass index (BMI) of all cases improved after treatment, compared with before treatment (*P* < 0.01). BMI of obese group reduced more significantly than that of nonobese group (*P* < 0.01), indicating that BBR was more applicable to prediabetes and the early stage of T2DM characterized by insulin resistance and obesity. More researches were needed, because BBR was merely the component of* RC* and did not represent* RC *totally. Based on large amount of clinical practice and modern researches, stagnancy, heat, deficiency, and damage are thought of as four stages of T2DM [66, 67]. Stagnancy and heat stages mostly appear in the early and middle stage of T2DM. Deficiency stage is equivalent to traditional Xiaokezheng, and damage stage often accompanies various complications. Deficiency and damage stages appear in middle and late stages.* RC* is commonly used in the heat and deficiency stages of T2DM [68]. Tang and Shen [69] observed the hypoglycemic efficacy of BBR on different TCM syndromes of T2DM; 120 patients were assigned to four groups, including syndromes of yin deficiency and excessive heat, damp-heat encumbering in the spleen, dual deficiency of qi and yin, and blood stasis in collaterals. BBR (0.5 g) was orally given three times daily for three months. Results indicated that BBR had better hypoglycemic effects on syndrome of damp-heat encumbering in the spleen than other groups; BMI decreased more significantly in group of damp-heat encumbering in the spleen (*P* < 0.05) than those of other groups (*P* > 0.05), which was consistent with properties of* RC*. The characteristics of splenic damp-heat syndrome commonly include bitter taste in the mouth, obesity, abdominal stuffiness and fullness, sticky fetid stool, yellow-greasy coating, and slippery and rapid pulse. [70] BBR could significantly improve the symptoms of splenic damp-heat syndrome of diabetic patients, as well as controlling the blood glucose effectively [71].

### 3.2. Reasonable Dose

The recommended dose of* RC* should be 15–45 g, even to a maximum dose of 60 g for alleviating the diabetic ketoacidosis (DKA) [72], whereas the routine dose in Chinese Pharmacopoeia (2010 edition) is 2–5 g is usually ineffective [73]. Liu [74] made a survey of the dose of* RC* in 1,321 effective formulas to treat T2DM (when the decrease percentage of fasting blood glucose (FBG) and postload plasma glucose (PBG) was >20% of those before treatment or the decrease percentage of Hemoglobin A1c (HbA1C) was >10% of that before treatment within 12 weeks, the formula was thought of as an effective formula, and other else was thought of as an ineffective formula), and the result showed that the common dose of* RC* to lower blood glucose was 15–45 g; the analysis on the correlation between dose of* RC* and level of FBG demonstrated that significant positive correlation existed, and the dose increased with the rise of FBG. The common dose of* RC* was 15 g when the level of FBG <7 mmol/L; 30 g when the level of FBG <10 mmol/L; and 30 g to 45 g when the level of FBG ≥10 mmol/L [68, 75, 76]. The dose of* RC* for treating T2DM was large; but the dose was small for treating the complications of T2DM [77]. Zhang et al. [78] observed the effects of Xinkai Kujiang Formula (XKF) with the different dose of* RC* on KKay mice with T2DM. XKF consisted of* RC* 20 g, Chaihu (*Radix Bupleuri*) 6 g, Huangqin (*Radix Scutellariae*) 10 g, Banxia (*Rhizoma Pinelliae*) 6 g, Wuweizi (*Fructus Schisandrae Chinensis*) 6 g, Dahuang (*Radix et Rhizoma Rhei*) 1 g, and Shengjiang (*Rhizoma Zingiberis Recens*) 3 g. The dose of* RC* was increased to 40 g in Xinkai Kujiang Jiawei Formula (XKJF) with doses of other herbs unchanging. Results showed that blood glucose decreased more significantly in XKJF group with dual doses of* RC *compared with that of XKF group (*P* < 0.05), indicating that the efficacy of XKF which lowered the blood glucose of KKay mice with T2DM was closely related to the dose of* RC*, and dose-effect relationship existed.

### 3.3. Variable Preparation Formulation

The therapeutic value of* RC* is mainly in the form of compound or polyherbal formulations and not as single herb. After referring to the documents, we found that several preparation formulations were mentioned to demonstrate hypoglycemic effects of* RC*, such as compound formula, decocting-free granules, BBR, total coptis alkaloids (TCA), pill, and tablet [17, 18, 65, 69, 75, 79, 80]. In general, decoction and decocting-free granules of* RC* are better than BBR and/or other alkaloids in terms of efficacy of lowering the blood glucose; the effect of decoction is better than that of pill and tablet; however the decoction is relatively unstable. Commonly speaking, compound formula decoction including* RC* is used for patients with high level of blood glucose, while pill and tablet are suitable for the control in stable level of blood glucose and long-term use, but this conclusion needs to be verified by more researches.

### 3.4. Toxicity/Side Effects and Solutions to Its Adverse Actions

According to the regulations of Singapore government in 1976,* RC* and BBR were forbidden to be used because it was deemed that a shortage of glucose 6 phosphate dehydrogenase (G6PD) could be caused after a pregnant woman or a newborn baby takes* RC* and BBR, which may lead to hemolytic jaundice of the newborn baby [81]. Liao [82] carried out a study where he provided* RC* decoction to 22 newborn babies in hospital, among which three were short of G6PD. According to his observation, the intake of* RC* would not cause hemolytic jaundice or any other side effects for either newborn babies that were short of G6PD or normal ones. Yi et al. [83] evaluated the toxicity of the* RC* and* RC* alkaloids (BBR, COP, PAL, and epiberberine). The cytotoxicity showed that the IC50 values of BBR, COP, PAL, and epiberberine in 3T3-L1 cells were 41.76, 56.48, 84.32, and 104.18 *μ*g/mL, which in HepG2 cells were 48.17, 64.81, 112.80, and 120.58 *μ*g/mL, respectively. In the acute toxicity assay, median lethal dose (LD50) values of four alkaloids were 713.57, 852.12, 1533.68, and 1360 mg/kg, respectively, which suggested that the toxicity of BBR was the maximum and PAL was the minimal. However, in the subchronic toxicity study, the currently recommended doses of* RC* alkaloids and* RC* consumed were relatively safe. There was also no abnormality in clinical signs, body and organ weights, hematological parameters, gross necropsy, and histopathology in mice after the oral administration of* RC *alkaloids and* RC* treatment. Linn et al. [84] carried out a retrospective analysis on the phenomena that* RC* and Huangbai (*Cortex Phellodendri Chinensis*) could cause hemolysis: they provided 20 patients with* RC* and Huangbai (*Cortex Phellodendri Chinensis*) for, respectively, 1,055 days and 1,252 days, demonstrating no organ toxicity or electrolyte disorder caused by* RC*. Therefore, they concluded that the use of* RC* within the dose range was safe and would not cause jaundice or kernicterus. Lee et al. [85] evaluated the no-observed-adverse-effect level (NOAEL) and the toxicity of* RC*, following repeat oral administration to rats for 13 weeks.* RC* was administered by oral gavage to groups of rats (*n* = 10/group) at dose levels of 0 (control), 25, 74, 222, 667, or 2000 mg/kg/day 5 times per week for 13 weeks, which suggested that the NOAEL of* RC* is determined to be 667 mg/kg/day for males and 2000 mg/kg/day for females. Qiu et al. [86] carried out a study on commonly used bitter-cold herbs and found that an intake of decocted* RC* liquid of the mice with a dose of more than 3 g·kg^−1^ could cause their death, the measured LD50 of* RC* being 4.89 g·kg^−1^. In addition, they found that* RC* had a direct effect to gastrointestinal tract and could cause loose stools and diarrhea. Later, they also found that bitter-cold herbs may do harm to the barrier function of gastric mucosa [87]. According to the animal experiment by Li and others [88], after the mice had taken a compatibility of* RC* and Huangqin (*Radix Scutellariae*) with a proportion of 1 : 0.5, the measured LD50 was equivalent to 20 times of that of an adult's daily dose of* RC*, and the measured LD50 under a combination of* RC* and Gancao (*Radix et Rhizoma Glycyrrhizae) *with the same proportion was equivalent to 42 times; when the proportion changed into 1 : 1 or 1 : 2, none of the mice died 7 days later. This proved that a compatibility of* RC* and other herbs such as Huangqin (*Radix Scutellariae*) or Gancao (*Radix et Rhizoma Glycyrrhizae*) could alleviate side effects of the mice and the optimal proportion should be 1 : 1 or 1 : 2. Chang et al. [89] retrospectively studied 116 patients with obese T2DM that were treated by the method of dispersing stagnation and clearing heat (including* RC*) without using hypoglycemic drugs; both of the 53 patients who were treated for one year and the 63 patients who were treated for two years showed very limited adverse reactions, and only 12 of them had got gastrointestinal reactions, including gastrointestinal discomforts and distention. These reactions alleviated when bitter-cold herbs reduced. If* RC *is taken over long period or used with large dose, it may cause “impairment of the stomach due to cold and bitterness,” which mainly means that it may damage yang qi (the yang aspect of qi, particularly referring to that aspect of qi as functional activities) in the middle* jiao* (middle energizer, namely, the upper abdominal cavity, i.e., the portion between the diaphragm and the umbilicus housing the spleen, stomach, liver, and gallbladder), marked by diminished function of the spleen and stomach in digestion and absorption [90], thus resulting in discomforts of gastrointestinal tracts. After repeatedly clinical practices, we solve the contradiction of using* RC* by means of compatibility of medicinals. The compatibility of formulas and medicinals is a feature of TCM, which may greatly decrease the adverse reactions caused by* RC* [88]. In clinical practice,* RC* is commonly combined with some warm and acrid herbs, because warm and acrid medicine could restrain cold and bitterness; moreover, the compatibility of warmth & acridity and cold & bitterness could promote the function of the spleen and stomach and harmonize the middle energizer [91]. Ganjiang (*Rhizoma Zingiberis*) or Shengjiang (*Rhizoma Zingiberis Recens*) is commonly combined with* RC *in the clinical practice; the regular proportion of* RC* to Ganjiang (*Rhizoma Zingiberis*) is 6 : 1 and the regular proportion of* RC* to Shengjiang (*Rhizoma Zingiberis Recens*) is 4 : 1. When the patient had weak function of spleen and stomach, the dose of Ganjiang (*Rhizoma Zingiberis*) or Shengjiang (*Rhizoma Zingiberis Recens*) can be increased; the proportion of* RC *to the ginger may be 2 : 1, even 1 : 1 [68, 75, 76].

## 4. Summary

The rapidly increasing DM is becoming a predominant healthy problem, affecting 92.4 million persons in China. With increasing incidence of obesity, T2DM is likely to become even more prevalent in the future. It has a significant impact on the quality of life and the number of death as well as on the financial resources of public health care system. The treatment of T2DM and its complications mainly depend on western hypoglycemic drugs, and/or insulin, but more and more patients have been concerned about the potential toxicity and side effects, and they failed to delay the progression of diabetic complications; sometimes the clinical efficacy is far from satisfactory. Due to positive views of patients towards complementary and alternative medicine (CAM) therapies, and the increased availability of them, CAM therapies are increasingly and frequently used globally [92–94]; the commonly used therapies are traditional Chinese medicines, acupuncture, nutritional supplements and advice, spiritual healing, and relaxation techniques [94].

Natural plants, especially Chinese herbal medicines, have built up a characteristic medical system directed by traditional Chinese medical theory and provided rational means for various diseases including DM [12].* Rhizoma coptidis*, a kind of classical heat-clearing and detoxifying herb, is playing an important role in treating T2DM, thus arousing strong interests in the mechanisms of its hypoglycemic activity. In this review, on one hand, we provided scientific evidence about the effective components, pharmacological researches, toxicity, and the randomized controlled trials (RCTs) on effectiveness and safety of* RC*. The modern investigation on* RC* pharmacological activity is actually developing and numerous scientific evidences are actually in progress. In recent years, some researches [95] found that diabetic patients have more Firmicutes and less Bacteroidetes than lean control; the change of gut microbiota caused activation of a network of inflammatory signal pathways via the Lipopolysaccharides (LPS) and CD14/toll-like receptor-4 (TLR4-) dependent pathway, which made the body in a state of low-grade inflammation; ultimately, T2DM came into being [96, 97].* RC* was demonstrated to treat T2DM possibly via modulating the composition of gut microbiota (enrichment of beneficial microbiota and inhibition of harmful microbiota) [98]. However, almost all of the researches are made based on its single component BBR or others;* RC *that contains various active components may be more effective than its single component BBR and could provide multiple therapeutic effects. More clinical trials and animal experiments are still needed to study* RC*. Several researches on compound herbal medicines are not acceptable for western people; one way to change this situation is standardization; the need for adequate standards of herbal medicines to ensure quality, safety, and efficacy should be highlighted.

On the other hand, Chinese herbal medicines, instead of component, are often used by Chinese doctors; we introduced traditional Chinese medical knowledge to western people make them understand more traditional Chinese medical theory.* RC* is herb of bitter flavor and cold property. In TCM theory, bitter flavor is in direct opposition to sweet flavor, so the bitter flavor is an excellent approach to counteract sweet flavor [99].* RC *was more applicable to prediabetes and the early stage of T2DM characterized by insulin resistance and obesity* RC* had better hypoglycemic effects on syndrome of damp-heat encumbering in the spleen, which may be related to the significant improvement of the symptoms of splenic damp-heat syndrome, such as bitter taste in the mouth, obesity, abdominal stuffiness and fullness, sticky fetid stool, yellow-greasy coating, and slippery and rapid pulse. Several preparation formulations on* RC *were mentioned, including decoction, decoction-free granules, pill, and tablet, and the therapeutic value of* RC* in market is mainly in the form of compound or polyherbal formulations and not as single herb. The common dose of* RC* to lower blood glucose was 15–45 g. Besides, the dose of* RC* for treating T2DM was relatively large; but the dose was small for treating diabetic complications. Most of these conclusions were based on clinical experience, the lack of scientific and experimental evidence was a problem, or the grade of evidence was low; several limitations existed in this review. More clinical trials and animal experiments are required to provide stronger evidence.

## Abbreviations

DM: Diabetes mellitus
TCM: Traditional Chinese medicine
*RC*: * Rhizoma coptidis*
T2DM: Type 2 diabetes mellitus
GLP-1: Glucagon-like protein-1
AMPK: Adenosine monophosphate-activated protein kinase pathway
InsR: Insulin receptor
PPARs: Peroxisome proliferator-activated receptors
InsR: Insulin receptor
LDLR: Low-density lipoprotein receptor
ERK: Extracellular signal-regulated kinase
HFD: High-fat diet-fed
BBR: Berberine
COP: Coptisine
PAL: Palmatine
JAT: Jatrorrhizine
HFHC: High-fat and high-cholesterol diet
HMGCR: 3-Hydroxy-3-methyl-glutaryl-CoA reductase
CYP7A1: Cholesterol 7-alpha hydroxy-lase
I/R: Ischemia and reperfusion
IL: Interleukin
TNF-*α*: Tumor necrosis factor-*α*
Rho: Rhodopsin
ROCK: Rho-kinase
ESI-MS: Electrospray ionization tandem mass spectrometry
IC50: Half maximal inhibitory
AR: Aldose reductase
RLAR: Rat lens aldose reductase
HRAR: Human recombinant aldose reductase
T1DM: Type 1 diabetes mellitus
AUC: Area under curve
*C*_max⁡_: Peak concentration
IC50: Half maximal inhibitory
STZ: Streptozotocin
P-GP: P-Glycoprotein
BMI: Body mass index
DKA: Diabetic ketoacidosis
FBG: Fasting blood glucose
PBG: Postload plasma glucose
HbA1C: Hemoglobin A1c
TCA: Total coptis alkaloids
G6PD: Glucose 6 phosphate dehydrogenase
LD50: Median lethal dose
NOAEL: No-observed-adverse-effect level
G6PD: Glucose 6 phosphate dehydrogenase
CAM: Complementary and alternative medicine
RCTs: Randomized controlled trials.
